# Aligning academia and industry for unified battery performance metrics

**DOI:** 10.1038/s41467-018-07599-8

**Published:** 2018-12-10

**Authors:** Zhan Lin, Tiefeng Liu, Xinping Ai, Chengdu Liang

**Affiliations:** 1 School of Chemical Engineering and Light Industry, Guangdong University of Technology, Guangzhou, 510006 P. R. China; 2 Hubei Key Lab of Electrochemical Power Sources, College of Chemistry and Molecule Science, Wuhan University, Wuhan, 430072 P. R. China; 3 Zhejiang Provincial Key Laboratory of Advanced Chemical Engineering Manufacture Technology, College of Chemical and Biological Engineering, Zhejiang University, Hangzhou, 310027 P. R. China

## Abstract

Exceptional performance reported for battery materials and devices in the scientific literature is often measured under conditions that are not aligned with practical applications. Aiming to bridge the gap between academia and industry, this Comment advocates the best practices for gauging performance and proposes guidelines on measurements with respect to a list of key metrics such as capacity, cyclability, Coulombic efficiency and electrolyte consumption.

## Introduction

The growing momentum behind the implementation of electric vehicles (EVs) and other renewable electricity generation technologies brings enormous opportunities as well as challenges to the energy industry^1,2^. This is particularly true for rechargeable batteries, such as lithium-ion batteries (LIBs), which are viewed as the most promising power source for EVs^3^. If EVs are to compete with fossil-fueled cars, they must deliver a driving range of ~800 km on a single charge^4^. However, modern LIBs only possess a gravimetric energy density of ~250 Wh kg^−1^, which is equivalent to 440 km for an EV loaded with a battery pack weighing 900 kg. Introducing more cells can certainly extend the driving distance; however, the total weight and the associated costs are prohibitive^5^. Thus, a more reasonable option is to adopt batteries with improved energy densities, e.g. 500 Wh kg^−1^, under which an EV can travel as far as 800 km on a single charge. Notably, the US has recently announced “Battery500 Consortium” (https://energystorage.pnnl.gov/battery500.asp), which is an initiative encouraging innovations in developing high energy density batteries for future needs^6^.

For a typical LIB cell, its energy density (*E*) is determined by the electrochemical voltage (*V*) between the utilized redox couples and the specific capacity (*C*) of the electroactive materials in the electrodes (Eq. 1)^7^,

1 $$E = \frac{{C_c \times C_a}}{{C_c + C_a}} \times (V_c - V_a)$$

where *C*_*c*_*/C*_*a*_ is the specific capacity of cathode/anode and *V*_*c*_/*V*_*a*_ is the electrochemical potential of cathode/anode.

**Fig. 1:**
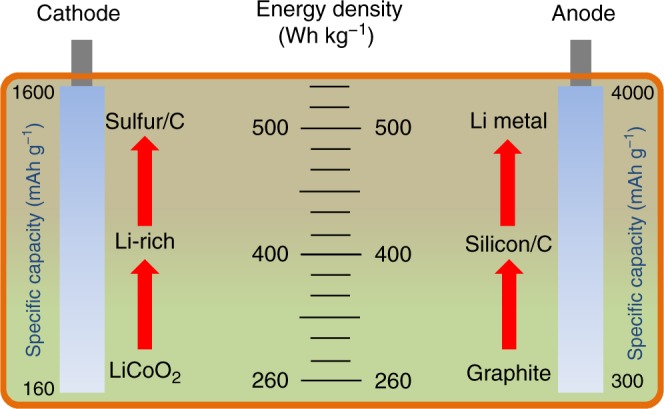
Specific energy densities of LIBs based on different cathode and anode materials. Data adapted from ref. ^7^

From Eq. 1, it becomes clear that the energy density of LIBs can be increased by increasing the operating voltage, or the charge-storage capacity, or the mass loading, or any combination. To this end, a variety of battery chemistries^8–12^ has been reported in recent years and Fig. 1 shows the corresponding energy densities. It can be seen that replacing the traditional graphite anode with silicon/carbon can double the energy density from 372 mAh g^−1^ to ~600–800 mAh g^−1^. Coupling a Li-rich cathode with the same silicon/carbon composite anode delivers a value of >400 Wh kg^−1^. Adopting a lithium-sulfur (Li-S) cell would yield an energy density approaching the target of 500 Wh kg^−1^.

Despite these claimed successes in increasing the energy density of LIBs, there are growing concerns in the community that the exceptional performance reported in academic research is increasingly inaccessible to practical applications^13^. This is because the optimization of one performance metric usually comes at the cost of losses in other parameters and the assessment itself tends to be based on experimental conditions that do not make sense for practical use. Herein, we examine the differences in experimental settings for both academia and industry, stressing that although academic and industrial research serve different purposes, for a better overall assessment of the battery materials and devices, there should be reconciliation in the evaluation of performance metrics.

## All performance metrics matter

**Fig. 2:**
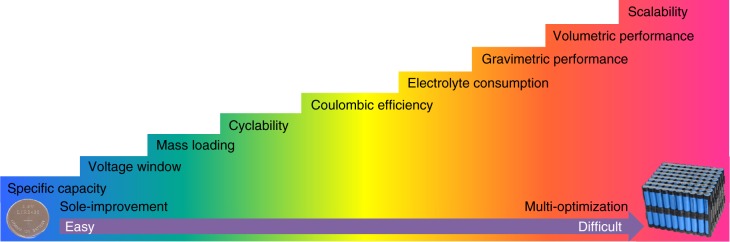
Various performance metrics of electrodes in LIBs from coin cells to battery packs

As shown in Fig. 2, the assessment of the performance of batteries requires the measurement and quantification of a series of performance metrics^14^, including specific capacity, voltage window, mass loading, cyclability, Coulombic efficiency, electrolyte consumption, gravimetric performance, volumetric performance and scalability. Reporting the performance based on a limited number of metrics does not give a realistic picture of the performance required by practical use.

### Specific capacity

Specific capacity is an indicator to the amount of electric charge stored by the electroactive materials in a unit mass. An important rule is that when calculating the value of specific capacity, both redox active and inactive materials in electrodes must be taken into account. Usually, its measurement is done by galvanostatic charge/discharge at a given potential window and under a certain temperature. Thus, the preparation and measurement conditions both influence specific capacity.

### Voltage window

The voltage window describes the potential range from the charging upper limit to the discharging lower limit. No standard procedure is available for the determination of its value. Given that a cathode with a high voltage platform and an anode with a low voltage platform are a favored combination, it is recommended that the discharging lower limit is at ~1.5–2.0 V in the cathode, while the charging upper limit should be below 1.5 V in the anode. Meanwhile, the voltage window of electrolyte must be electrochemically stable.

### Mass loading

Mass loading is defined by the weight of electrode slurry on the current collector in a unit area. In a lab cell, an electrode is often coated with low mass loading (below 2 mg cm^−2^)^15^. Such a thin electrode laminate (<20 μm) reduces electrical pathways and favors electrolyte infiltration, ensuring negligible potential polarization for maximum performance investigation. As areal loading increases, the electrode film becomes thicker. Thick electrodes tend to fracture and delaminate from the current collector after coating and drying, making high-loading electrodes more difficult to produce. In current LIBs, the electrode is prepared with high mass loadings of ~5–10 mg cm^−2^, allowing for an areal capacity of ~3–4 mAh cm^−2^. For next-generation LIBs with 500 Wh kg^−1^, the areal capacity should increase to ~6–7 mAh cm^−2^. Such high areal capacity can only be addressed by combining high-capacity material with high mass loading. Therefore, high mass loading in the electrode should be a focus for high energy density LIBs.

### Structural stability

Cyclability is a measure of the number of times the electrode material can sustain its initial capacity during cycling. Galvanostatic charge/discharge is the standard method for evaluating the cycling stability. Excellent cyclability requires (i) electrode materials that possess high structural stability against electrochemical strain and volume fluctuation, and (ii) a stable interface between electrolyte and electrode, enabling reversible ion transfer in each cycle without lithium loss. The former involves the nature of active materials such as its crystalline structure, while the latter is related to the Coulombic efficiency (CE).

### Coulombic efficiency

An ideal CE is 100%, indicating that all the lithium ions leaving the cathode in a fully charged state can return to the cathode in a fully discharged state. Some lithium, however, is consumed in every cycle, trapped in the formation of solid electrolyte interface (SEI) due to side reactions. Thus, the CE is less than 100% for each cycle. Table 1 shows the influence of CE on capacity retention in a full cell. We assume that the mechanical properties of electroactive materials do not affect cycling performance. When the CE is 99%, the remaining lithium after 20 cycles is only 0.99^20 ^= 81.79%, indicating ~20% loss of lithium. After 200 cycles, full LIBs only deliver 13.4% of their initial capacity and thus cannot be used anymore. Normally, capacity retention of 80% is a criterion for the life of an energy storage device for the EV industry. Therefore, CE of 99.96% is required for cycling stability up to 500 cycles for commercialization.

**Table 1:** Influence of Coulombic efficiency (CE) on capacity retention in a theoretical full cell

CE cycle	99%	99.8%	99.9%	99.96%	99.98%
20	0.99^20^ = 81.79%	96.08%	98.02%	99.20%	99.60%
100	36.60%	81.86%	90.48%	96.08%	98.02%
200	13.40%	67.01%	81.86%	92.31%	96.08%
500	0.66%	36.75%	60.64%	81.87%	90.48%
1000	0.00%	13.51%	36.77%	67.02%	81.87%

### Gravimetric energy density

Gravimetric energy density reflects how much electric charge can be stored in a unit mass of material/device. Academic research tends to use a large excess of counter electrode and electrolyte in the cell. As a result, the obtained performance does not necessarily represent a realistic case. In industry, all parameters including the mass ratio, anode and cathode loading, and the amount of electrolyte must be considered.

### Volumetric energy density

As opposed to gravimetric energy density, the volumetric metric gauges the energy density in a unit volume and is more difficult to calculate because the electrode density is jointly determined by redox active and inactive materials. To increase volumetric energy density, the amount of inactive materials must be minimized so as to allow the incorporation of more electroactive materials into a fixed electrode volume. Particularly, the amount of electrolyte should be reduced to a level that does not compromise electrochemical performance in the electrode. If volumetric energy density of the LIB electrode is calculated using reliable electrode parameters and performance, we can usually divide this value by a factor of 4–5 to extrapolate expected LIB volumetric performance^14^.

## Impacts of experimental settings on performance metrics

For a clear understanding of the impact of experimental parameters on performance metrics, several specific examples are presented below. Evaluating performance metrics in academic research is the subject in highly idealized process streams. Practically viable LIBs should seek to understand the trade-offs that exist among these multiple metrics.

### Polarization of high mass loading

**Fig. 3:**
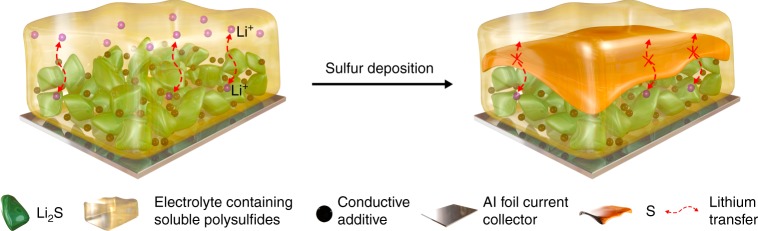
Sulfur deposition and the resulting blocking causing failure of a high-loading sulfur cathode

We take Li-S batteries as an example, which still have many challenges to overcome, such as limited cycle life, high self-discharge rates and overheating at the end of charge, the shuttle effect, the insulating nature of sulfur, and the huge volume change (76%) during cycling^9^. Enormous efforts have been devoted to identify strategies and methods to tackle these issues. These solutions might be useful concerning that test conditions are not applicable to industry or built on metrics that do not really matter for practical applications. For practical Li-S batteries with an energy density >500 Wh kg^−1^, the mass loading of sulfur needs to reach ~7–8 mg cm^−2^ or even higher^15^, together cooperating with a metallic lithium anode and a small amount of electrolyte. To achieve such a high mass loading we need a thick sulfur electrode (>300 µm), which can lead to serious polarization of the electrode oriented to the electrolyte. Polysulfides are preferably reduced to sulfur on the electrode surface, which causes nonhomogeneous deposition and blocking of the internal porous structure, potentially ending in accidental electrochemical deactivation of the sulfur cathode as a “sudden death”. Figure 3 describes the sulfur deposition blocking a high-loading sulfur cathode. This complicated process rarely occurs in lab research as the sulfur cathode is controlled under low mass loading.

### Insufficient Coulombic efficiency during cycling

Large volume changes of Si during lithiation and de-lithiation processes are the main origin of insufficient cycle life in Si anodes, specifically causing particle pulverization, electrode fracture from the current collector, and the growth of SEI film^16^. As mentioned previously, two factors determine the cycling stability of the electrode, namely the nature of active material and the value of CE in each cycle. Although many approaches to maintain material and electrode integrity have effectively improved the cycling lifespan of Si anodes, the CE is still far from practical application requirements. Similar issues occur for Li-S batteries. However, low CE which results in poor cycling performance is masked by the half-cell configuration using a lithium metal anode. Lithium metal disks (15.6 mm × 0.55 mm) with a capacity of >200 mAh, which far exceeds that of low-mass-loading electrodes (~1–2 mAh), can constantly offset lithium loss during cycling. In addition, excess electrolyte also enables long cycling of electroactive materials without considering potential decomposition of electrolyte. High CE with >99.9% has not as yet attracted much attention in the literature. These auxiliary data are still not directly applicable to industrial technologies.

### Oversimplified energy density

It is tempting to oversimplify the energy density of LIBs. In recent years, substantial efforts have focused on improving gravimetric specific capacity and rate performance. One effective strategy is the design and synthesis of porous material/electrode architecture (e.g. hierarchical, core-shell, sandwich-like, and array nanostructures). Such a porous structure not only offers the pseudo-capacity from high specific surface area, but is also favorable for fast diffusion of lithium ions through electrolyte infiltration^17^. With regards to practical application, electroactive materials with hierarchical architectures have low tap density, making them unlikely to meet volumetric energy density demands. For example, the tap density of Si-based anode ranges from 0.4 to 1.0 mg cm^−3^. Thus, its volumetric energy density would be much lower than a graphite anode with an electrode density of ~1.4–1.8 mg cm^−3^^16^. Calendering the electrode surely increases volumetric energy density, but hierarchical architectures are inevitably destroyed at high pressures, resulting in the loss of their electrochemical behavioral advantages.

### Different electrode fabrication

Lab and commercial electrodes differ sharply with respect to the component ratio between electroactive and inactive materials. In the lab, reports of exceptional electrode capacity often depend heavily on large amounts of conductive additives, binders and electrolyte^18^. However, this does not create an energy-dense LIB electrode. To implement high-capacity electroactive materials for high energy density LIBs, some parameters have been provided in recent studies. In research on Li-S batteries, for example, key parameters including high weight percentage of sulfur (>90%), low electrolyte/sulfur ratio (<1.9 mL g S^−1^), and limited lithium excess (≈50–100%) have been considered^19^. A further development of the mechanical property, electrochemical performance, and energy density calls for a series of optimizations and balances in this effort.

Except for mass loading, CE, energy density, and fabrication process in LIBs, other performance metrics, such as thermal stability, scale cost, electrolyte consumption as well as material recyclability occur in the gap between academia and industry, challenging industrial or practical uptake of new technologies. As a result, electroactive materials accessible to practical applications must undergo multi-objective optimization. A solution-oriented research for practical LIBs should use industrial parameters and process novel designs from the earliest stage.

### The way forward

The success of “Battery500 Consortium” will be critically dependent on the progress in the development of high-capacity electroactive materials toward practically viable performance metrics. The gap between academia and industry challenges the actual use of these materials and related battery chemistry, even resulting in the failure and abandonment of what might at first be considered promising technologies. Bridging this gap requires that academic research turn to improve performances under industrial requirements, such as high mass loading, limited electrolyte, and excellent CE. Three decades of work on LIBs has provided an enormous body of knowledge for performance metrics upgrading from academia to industry. How do we harness this opportunity? Firstly, fundamental science can help by revealing underlying chemical and structural mechanisms of high-capacity electroactive materials by in situ technologies probing the reaction during electrochemical cycling. For example, Cui and co-workers first used cryopreservation electron microscopes to analyze SEI film^20^. This basic research is key to comprehensively understanding the formation and evolution of SEI film during cycling, which is not usually done by industry. A stable SEI layer not only favors the anode cycling, but also enables high voltages. Secondly, standard protocols between academia and industry should be established. Any information involving electrode preparation and measurement must be clearly described, including respective ratios (or weight percentage) among active material, conductive additive, and binder in the slurry, areal mass loading coating on the current collector, voltage window, and ambient temperature. Recently, academic research has begun to provide the additional results related to high loading electrode, such as Si anode^21^ and sulfur cathode^18^, inspecting the practicability of proposed methods. Although maintaining cycle life and high capacity for LIBs under industrial parameters are very challenging, such results can reflect real case of as-prepared electrodes in practical applications, which is of value to industry. Thirdly, sharing experience and knowledge between scientists and engineers will continue to be vital when solving key problems in LIBs. Effective communications between the two sides could accelerate the transformation of lab-scale innovations to industrial applications. Therefore, we recommend the reconciliation of battery performance metrics to link academia and industry, and believe that such reconciliation will hasten large-scale commercialization of high-capacity electroactive materials for high-energy-density rechargeable batteries in the near future.

## References

[1] Chu S, Majumdar A (2012). Opportunities and challenges for a sustainable energy future. Nature.

[2] Obama B (2017). The irreversible momentum of clean energy. Science.

[3] Crabtree G (2015). Perspective: the energy-storage revolution. Nature.

[4] Noorden RV (2014). A better battery. Nature.

[5] Bourzac K (2015). Batteries: 4 big questions. Nature.

[6] Armand M, Tarascon JM (2008). Building better batteries. Nature.

[7] Yang Y (2010). New nanostructured Li_2_S/silicon rechargeable battery with high specific energy. Nano Lett..

[8] Lee J (2018). Reversible Mn^2+^/Mn^4+^ double redox in lithium-excess cathode materials. Nature.

[9] Ji X, Lee KT, Nazar LF (2009). A highly ordered nanostructured carbon-sulphur cathode for lithium-sulphur batteries. Nat. Mater..

[10] Chan CK (2008). High-performance lithium battery anodes using silicon nanowires. Nat. Nanotechnol..

[11] Qian J (2015). High rate and stable cycling of lithium metal anode. Nat. Commun..

[12] Su YS, Manthiram A (2012). Lithium–sulphur batteries with a microporous carbon paper as a bifunctional interlayer. Nat. Commun..

[13] Walton KS, Sholl DS (2017). Research challenges in avoiding “showstoppers” in developing materials for large-scale energy applications. Joule.

[14] Balducci A, Belanger D, Brousse T, Long JW, Sugimoto W (2017). Perspective—A guideline for reporting performance metrics with electrochemical capacitors: from electrode materials to full devices. J. Electrochem. Soc..

[15] Lv D (2015). High energy density lithium–sulfur batteries: challenges of thick sulfur cathodes. Adv. Energy Mater..

[16] Jin Y (2017). Self-healing SEI enables full-cell cycling of a silicon-majority anode with a coulombic efficiency exceeding 99.9%. Energy Environ. Sci..

[17] Wang Q, Yan J, Fan Z (2016). Carbon materials for high volumetric performance supercapacitors: design, progress, challenges and opportunities. Energy Environ. Sci..

[18] Liu J (2017). Exploiting a robust biopolymer network binder for an ultrahigh-areal-capacity Li-S battery. Energy Environ. Sci..

[19] Peng HJ, Huang JQ, Cheng XB, Zhang Q (2017). Review on high-loading and high-energy lithium-sulfur batteries. Adv. Energy Mater..

[20] Li Y (2017). Atomic structure of sensitive battery materials and interfaces revealed by cryo–electron microscopy. Science.

[21] Xu Z (2018). Silicon microparticle anodes with self-healing multiple network binder. Joule.

