# Publisher Correction: Aligning academia and industry for unified battery performance metrics

**DOI:** 10.1038/s41467-019-08321-y

**Published:** 2019-01-15

**Authors:** Zhan Lin, Tiefeng Liu, Xinping Ai, Chengdu Liang

**Affiliations:** 1 School of Chemical Engineering and Light Industry, Guangdong University of Technology, Guangzhou, 510006 P. R. China; 2 Hubei Key Lab of Electrochemical Power Sources, College of Chemistry and Molecule Science, Wuhan University, Wuhan, 430072 P. R. China; 3 Zhejiang Provincial Key Laboratory of Advanced Chemical Engineering Manufacture Technology, College of Chemical and Biological Engineering, Zhejiang University, Hangzhou, 310027 P. R. China

Correction to: *Nature Communications*; 10.1038/s41467-018-07599-8; published online 10 December 2018

The original PDF version of this Article contained an error in Fig. 2, in which only the central part of the figure, between ‘Mass loading’ and ‘Gravimetric performance’, was visible. This has been corrected in the PDF version of the Article. The HTML version was correct from the time of publication.

